# Protein carbonylation as a modulator of fibrin clot properties in thyroid disorders: impact of therapy

**DOI:** 10.1007/s11239-025-03180-5

**Published:** 2025-09-26

**Authors:** Kamila W. Undas, Julianna Dąbrowa, Joanna Natorska, Piotr Mazur, Alicja Hubalewska-Dydejczyk, Anetta Undas

**Affiliations:** 1 Faculty of Medicine, Jagiellonian University Medical College, Krakow, Poland; 2 Faculty of Physics, Astronomy and Applied Computer Science, Jagiellonian University, Krakow, Poland; 3 Department of Thromboembolic Disorders, Institute of Cardiology, Jagiellonian University Medical College, 80 Pradnicka St. 31-202, Krakow, Poland; 4 Krakow Centre for Medical Research and Technologies, St. John Paul II Hospital, 80 Pradnicka St. 31-202, Krakow, Poland; 5 Department of Cardiovascular Surgery and Transplantology, Institute of Cardiology, Jagiellonian University Medical College, Krakow, Poland; 6 Department of Cardiac Surgery, University, Leipzig Heart Center, 39 Strümpellstr, 04289 Leipzig, Germany; 7 Chair and Department of Endocrinology, Jagiellonian University Collegium Medicum, Krakow, Poland

**Keywords:** Oxidative stress, Fibrin, Hyperthyroidism, Hypothyroidism, Protein carbonylation

## Abstract

Protein carbonylation (PC), a marker of oxidative stress, was shown to be elevated in both hyperthyroid and hypothyroid disorders. These conditions are associated with unfavorable fibrin clot properties. We sought to investigate whether elevated PC is associated with prothrombotic markers in hyperthyroid and hypothyroid individuals before and following effective therapy. We studied 31 hyperthyroid, 29 hypothyroid patients, and 29 sex- and age-matched controls. Along with plasma total PC content, we measured fibrin clot properties (fibrin clot permeability, K_s_; clot lysis time, CLT), fibrinolysis proteins, and thrombin generation before and after 3-month successful therapy. Hyperthyroid patients had a tendency to higher PC (+ 9.1%; *p* = 0.05), while hypothyroid individuals had 17.2% higher PC (*p* = 0.01) compared with controls, without any difference between the patient groups. Pre-treatment PC inversely correlated with K_s_ in both hyper- (*R*=-0.425, *p* = 0.017) and hypothyroid (*R*=-0.510, *p* = 0.005) individuals, while solely in hyperthyroid patients PC was associated with CLT (*R* = 0.556, *p* = 0.001), but not with fibrinolysis inhibitors, or other hemostatic markers. On-treatment PC, which decreased by 19.6% (*p* < 0.001) in hyperthyroid and by 23.4% (*p* < 0.001) in hypothyroid patients reaching the control levels, was associated with K_s_ (*R*=-0.401, *p* = 0.031) and CLT (*R* = 0.537, *p* = 0.003) only in the hypothyroid group. In hyper- and hypothyroid patients elevated PC may contribute to formation of more compact fibrin clot networks with impaired fibrinolysis in the former group. Reduced PC following thyroid hormone normalization maintained its impact on fibrin clot properties solely in hypothyroid patients, which indicates complex effects of oxidative stress on blood coagulation.

**Graphical abstract:**

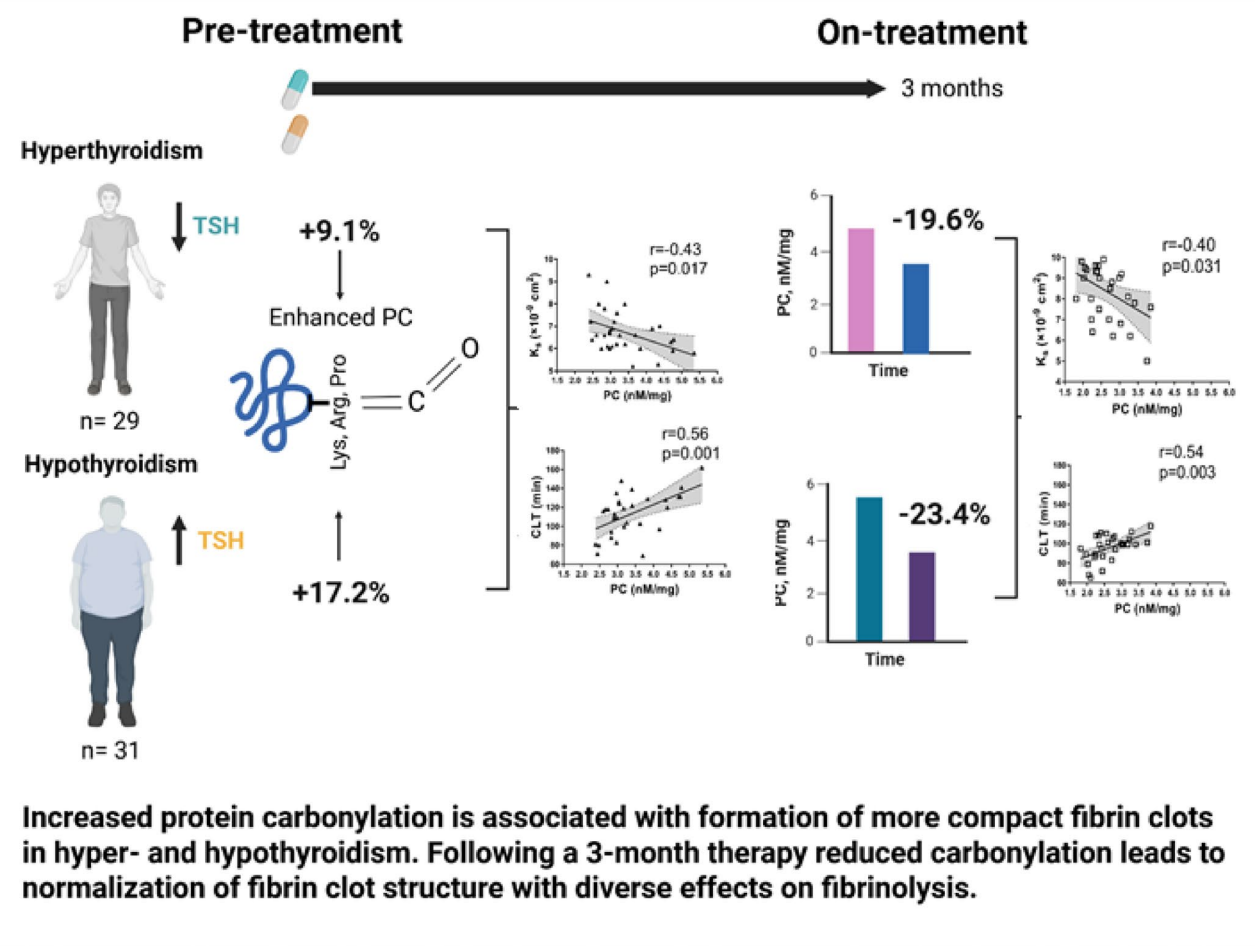

## Introduction

Protein carbonylation (PC) is a process involving covalent attachment of carbonyl groups to amino acid side chains [1]. Mechanisms underlying this post-translational modification involve oxidation of amino acid side [2], covalent binding of lipid peroxidation-derived aldehydes [3], and the formation of advanced glycation end products [4]. The post-translational modifications lead to functional changes in proteins by disrupting interactions between proteins, receptors, and enzyme inactivation [1, 2, 4].

Evidence indicates that fibrin clot properties and fibrinolysis are both affected by oxidative modifications of fibrinogen and other proteins [5–7]. Enhanced oxidative modifications of fibrinogen generally lead to the formation of more compact fibrin networks, composed of thinner fibers, and relatively resistant to lysis [5–8]. Carbonylated fibrinogen purified from patients' plasma has reduced susceptibility to plasmin-dependent degradation [9]. However, depending on the level of oxidative stress, oxidative modifications, including carbonylation, occur to a greater or lesser extent in other proteins involved in blood coagulation and fibrinolysis. Elevated PC was shown to be associated with impaired plasminogen binding to fibrin and prolonged clot lysis in patients with venous thrombosis [10]. It has been suggested that oxidative changes in plasma-purified plasminogen reported at 20 sites are associated with decreased fibrinolysis in patients [11]. The fibrin-binding affinity of tissue-type plasminogen activator (tPA) could also be decreased upon exposure to oxidants, but the catalytic activity of tPA and its binding to plasminogen are rather unaltered [12]. The impact of carbonylation occurring in vivo on all activators and inhibitors is poorly elucidated.

Elevated PC levels have been shown to be associated with the so-called prothrombotic fibrin clot phenotype, i.e., faster formation of denser, less permeable fibrin networks displaying impaired fibrinolysis, largely due to fibrinogen carbonylation, as demonstrated in acute ischemic stroke [13], coronary artery disease [14], and venous thromboembolism [10].

There is strong evidence that enhanced oxidative stress occurs in both hyperthyroidism [15] and hypothyroidism [16], involving the generation of reactive oxygen species (ROS), with enzymes such as nicotinamide adenine dinucleotide phosphate (NADPH) oxidase, myeloperoxidase, superoxide dismutase, and glutathione peroxidase [17]. However, less is known about PC as a specific aspect of oxidative stress in vivo, in thyroid disorders. In hyperthyroid individuals, excessive metabolic activity results in increased ROS production, leading to heightened PC and increased protein desialylation [18, 19]. Hypothyroidism is also characterized by increased PC content [20] and lipid peroxidation [21, 22]. Nevertheless, normalization of thyroid hormone levels significantly reduces oxidative stress [22].

Hyperthyroidism and hypothyroidism have been shown to be associated with increased risk of venous and arterial thromboembolism, with more potent evidence for the former disease [23, 24]. Moreover, patients with hypothyroidism more frequently demonstrated dyslipidemia [25] and increased cardiovascular risk [26]. Several mechanisms, including hyperfibrinogenemia and increased thrombin generation, contribute to the increased thrombotic risk in thyroid dysfunction [27, 28]. It has also been shown that elevated thyroid stimulating hormone (TSH) reduces fibrinogen, while higher free thyroxine (FT4) increases tPA [29, 30].

In 2014 it was demonstrated that hyperthyroidism and hypothyroidism are characterized by formation of more compact and poorly lysable fibrin clots, which improves following normalization of thyroid hormones [31]. Such unfavorably altered fibrin clot properties have been observed in patients at risk of thromboembolic events [32]. It is unclear whether PC is involved in alterations to fibrin clot phenotype observed in thyroid diseases.

We sought to investigate whether increased PC is associated with prothrombotic markers, particularly plasma fibrin clot structure and function, in patients with hyperthyroidism and hypothyroidism before and after normalization of thyroid function.

## Materials and methods

### Study population

We recruited 32 consecutive adult individuals with documented hyperthyroidism and 30 individuals with hypothyroidism in a tertiary center in Krakow, Poland. The current study group was a subset of the population described previously [31]. Hyperthyroidism was defined by a TSH concentration below 0.27 µIU/mL coupled with an FT4 concentration above 22 pmol/L and/or free triiodothyronine (FT3) concentration above 7.8 pmol/L; patients with low TSH and normal FT4 and FT3 were identified as having subclinical hyperthyroidism [31]. Among hyperthyroid subjects, Graves’ disease was confirmed if human thyroid receptor-stimulating antibodies (h-TRAB) were above 1.8 IU/L. For hypothyroidism, a diagnosis was made when TSH exceeded 4.2 µIU/mL in conjunction with FT4 concentration below 12 pmol/L. Individuals with TSH in the 4.3–10 µIU/mL range but normal FT4 were considered to have subclinical hypothyroidism. Furthermore, Hashimoto’s thyroiditis was diagnosed when anti-thyroid peroxidase (anti-TPO) and/or anti-thyroglobulin (anti-TG) antibodies were detected, using thresholds of 34 and 10 IU/mL, respectively. As many as 29 controls, matched for age and sex, were recruited from hospital personnel and their relatives.

We excluded patients on anticoagulant therapy, those with recent acute coronary syndrome, or venous thromboembolism and severe disease (malignancy, liver injury, chronic kidney disease), acute infection, or pregnant women. All participants provided written informed consent, and the study was approved by the Bioethical Committee.

Diabetes mellitus was identified either by ongoing insulin or oral hypoglycemic treatment, by fasting blood glucose > 7 mmol/L on two separate occasions, by hemoglobin A1C ≥ 6.5%, by blood glucose ≥ 11.1 mmol/L with typical hyperglycemia symptoms, or by blood glucose ≥ 11.1 mmol/L in a 2-hour sample in glucose tolerance test. Arterial hypertension was diagnosed based on a documented current antihypertensive treatment or repeated blood pressure ≥ 140/90 mmHg. Coronary artery disease (CAD) was defined by a history of acute myocardial infarction, coronary revascularization, or significant coronary stenosis on angiography.

Hyperthyroid individuals received either thiamazole, with doses adjusted biweekly or radioactive iodine (RAI) therapy at the physician’s discretion. Hypothyroid patients were treated with levothyroxine (LT4), with dosage modifications performed monthly. After a 3-month therapy, the patients were reassessed, including blood tests.

### Laboratory investigations

Venous blood samples were drawn from the antecubital vein using minimal tourniquet application after an overnight fast, between 8 and 10 AM. In patients with thyroid disorders, samples were collected twice at baseline and after 3 months of therapy; control subjects were sampled once. Routine assays were used to measure serum glucose, lipid profile, and creatinine. TSH, FT4, FT3, anti-TPO, and anti-TG were quantified using electrochemiluminescent immunoassays (Roche Diagnostics, Basel, Switzerland), and plasma h-TRAB levels were measured via a radioimmunometric assay (B.R.A.H.M.S, Hennigsdorf, Germany). Fibrinogen concentration was determined using the Clauss method. High sensitivity C-reactive protein (CRP) was measured by latex nephelometry (Siemens, Marburg, Germany).

Plasminogen was assessed using chromogenic assay (STA Stachrom plasminogen, Diagnostica Stago, Asnières, France). Levels of plasminogen activator inhibitor-1 (PAI-1) and tPA antigens were determined by ELISAs (Hyphen BioMed, Neuville-Sur-Oise, France), while PAI-1 activity was evaluated using a chromogenic assay (Chromolize PAI-1, Trinity Biotech, County Wicklow, Ireland). Thrombin-activatable fibrinolysis inhibitor (TAFI) antigen was measured by ELISA (Chromogenix, Lexington, MA, USA) and its activity was determined using a chromogenic assay with the ACTICHROME^®^ Plasma TAFI Activity Kit (American Diagnostica, Stamford, CT, USA). Peak thrombin was evaluated using calibrated automated thrombography (CAT; Thrombinoscope BV, Maastricht, the Netherlands) as described [31] .

### Protein carbonylation

The PC content was determined following the protocol by Becatti et al. [9]. In short, 400 µL of 2,4-dinitrophenylhydrazine was mixed with 100 µL of plasma. After allowing the mixture to incubate, trichloroacetic acid was added to precipitate the proteins. The resulting pellet was washed using an equal mixture of ethanol and ethyl acetate, then resuspended in 500 µL of guanidine hydrochloride. The PC content was determined using a molar extinction coefficient of 22,000 M⁻¹ cm⁻¹.

### Fibrin clot analysis

Plasma fibrin clot permeability (K_s_) was evaluated using a pressure-driven system. In this method, citrated plasma was mixed with calcium chloride (final concentration, 20 mmol/L) and human thrombin (final concentration, 1 U/mL) to induce clot formation. The clots were then connected via plastic tubing to a buffer-filled reservoir, and the volume of liquid passing through each clot over a set period was recorded. K_s_ was determined based on an equation incorporating variables such as flow rate, clot length, viscosity, cross-sectional area, and pressure difference. Lower K_s_ values indicate decreased clot permeability.

Fibrinolysis efficiency was assessed as previously described [33]. Lysis was induced by recombinant tPA (final concentration, 60 ng/mL, Boehringer Ingelheim, Ingelheim, Germany) and involved mixing citrated plasma with calcium chloride (final concentration, 15 mmol/L), human tissue factor (Innovin, Dade Behring, final concentration, 0.6 pmol/L ), and phospholipid vesicles (final concentration, 12 µmol/L), all diluted in Tris-buffered saline. The process was monitored spectrophotometrically at 405 nm and 37 °C, with clot lysis time (CLT) defined as the interval from the midpoint of clot formation to the stabilization phase indicating complete lysis. The investigations were performed by technicians blinded to the subjects’ clinical status.

Representative fibrin clots (*n* = 3) for each group were fixed using 2.5% glutaraldehyde, washed with distilled water, dehydrated in graded water-ethanol solutions, dried by the critical point procedure and sputter coated with gold. Samples were scanned in 10 different areas (microscope JEOL JCM-6000; JEOL Ltd., Tokyo, Japan) at a magnification of 5,000x to determine a mean fibrin fiber diameter of at least 100 fibers per clot, using the Image J software (US National Institutes of Health, Bethesda, MD, USA).

### Statistical analysis

Categorical variables were compared using Fisher’s exact test or χ^2^ test, as appropriate, with the Bonferroni correction in post hoc analyses. Shapiro-Wilk test and histogram analysis were employed to check for normal distribution of continuous variables. Data are presented as mean ± standard deviation if the parameter was normally distributed in all groups or median (interquartile range) if the variable was non-normally distributed in any group. Homogeneity of variance was checked using Levene’s test. To assess intergroup differences between hyper-, hypothyroid patients and controls, Kruskal-Wallis test with Dunn’s post hoc and one-way ANOVA with Tukey’s post hoc (for equal variance) or Games-Howell post hoc (for unequal variance) were used for non-normally and normally distributed continuous variables, respectively. FT3 and FT4 levels between hyper- and hypothyroid patients were compared using Mann-Whitney U test. For evaluation of differences in parameters before and after treatment, Wilcoxon signed-rank test or paired Student t-test was used for non-normally and normally distributed variables, respectively. Assessment of linear correlations between variables was conducted with Pearson’s correlation coefficient calculated if both variables were normally distributed and Spearman’s rank coefficient if any of the compared parameters were non-normally distributed. A two-sided p-value < 0.05 was considered statistically significant. Statistical analysis was performed using SPSS Statistics (IBM Corp. Released 2023. IBM SPSS Statistics for Windows, Version 29.0.2.0 Armonk, NY: IBM Corp).

## Results

In the final analysis, we assessed 31 hyperthyroid patients diagnosed with Graves’ disease and 29 hypothyroid patients with Hashimoto disease since one subject in each group was excluded due to incomplete data (Table 1). Five (16.1%) hyperthyroid patients and 8 (27.6%) hypothyroid individuals had subclinical disease. None of the patients had prior venous thromboembolism. The three groups did not differ in terms of demographic and clinical variables, except for a slightly higher BMI in the hypothyroid group as compared to hyperthyroid individuals (Table 1). None of the patients took oral contraceptives or menopausal hormonal therapy.

### At baseline

**Fig. 1:**
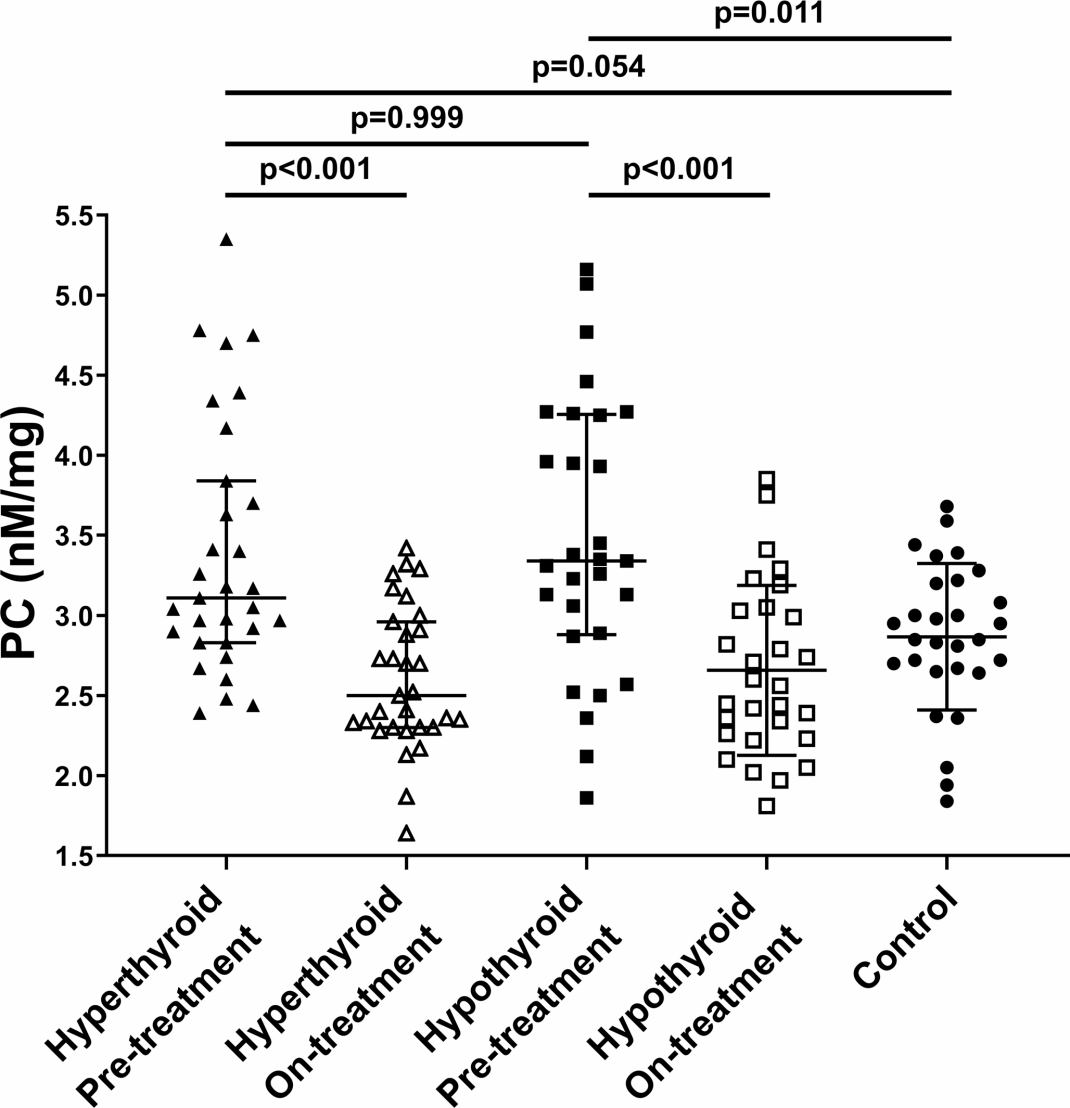
Dot plot of protein carbonylation (PC) levels in control, hypothyroid and hyperthyroid groups before and after treatment compared with healthy controls

There was a non-significant trend of higher PC (9.1%) in the hyperthyroid individuals compared to controls (*p* = 0.054), while median PC levels were 17.2% higher in the hypothyroid group compared to controls (*p* = 0.011) (Fig. 1). In hypothyroid individuals, but not in those with hyperthyroidism, PC levels correlated with age (*R* = 0.431; *p* = 0.019). No associations of PC with sex, BMI, smoking or comorbidities were found in any group (all *p* > 0.05). Pre-treatment PC levels showed no associations with any thyroid hormones or other disease markers.

#### Fibrin clot permeability

Fibrin clot phenotype analysis showed that patients with hyperthyroidism had 12% (*p* = 0.001) lower K_s_ compared with controls (Table 1). Among hypothyroid individuals in comparison to the control group, we observed no differences in K_s_ (Table 1). Hyper- and hypothyroid patients had comparable K_s_ prior to therapy.

**Fig. 2:**
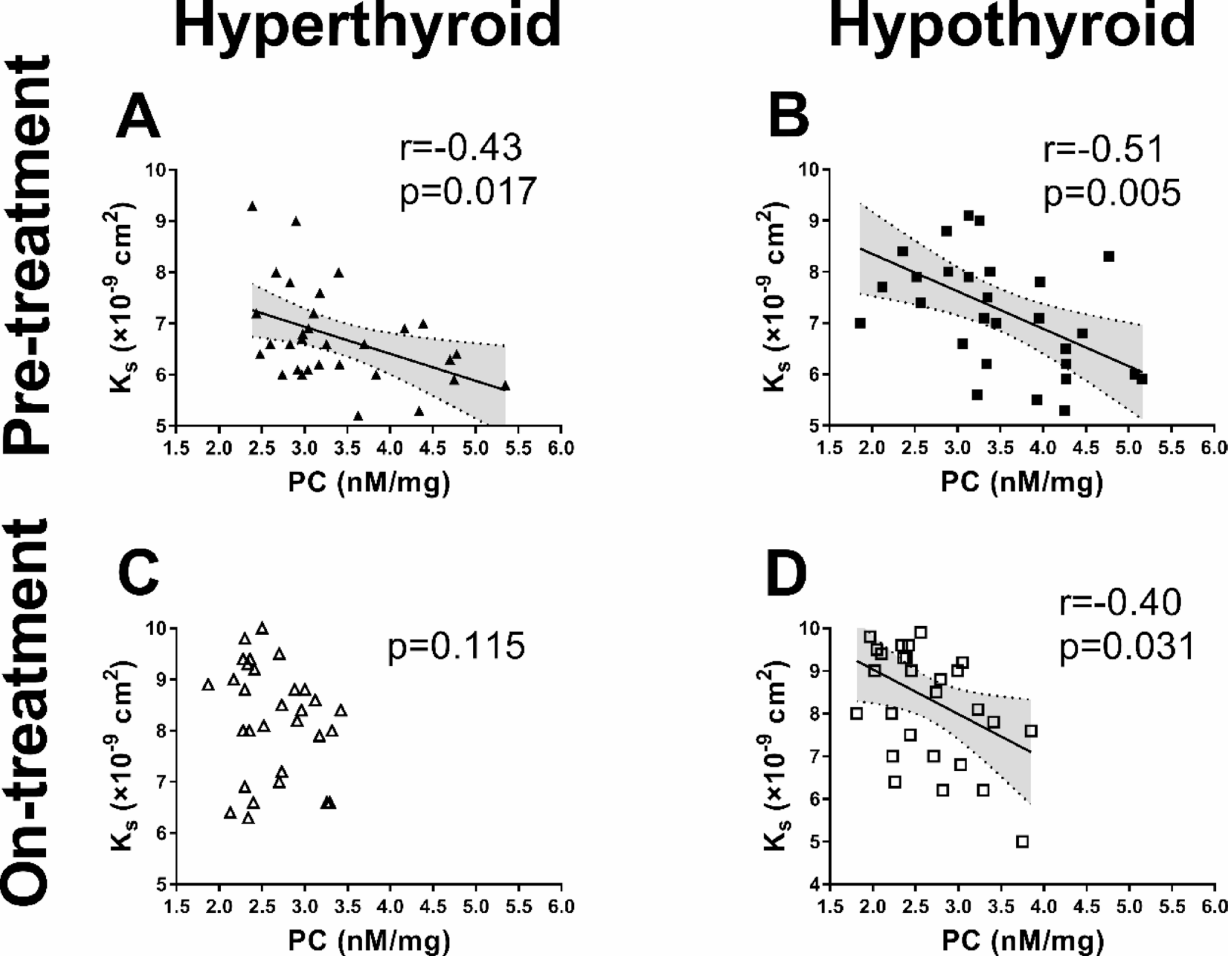
Correlations of protein carbonylation (PC) with permeation coefficient (K_s_) in hyperthyroid and hypothyroid patients before and on treatment

At baseline in both hyperthyroid and hypothyroid patients PC inversely correlated with K_s_ (Fig. 2A and B), despite no associations with fibrinogen in this group (*p* > 0.05).

**Fig. 3:**
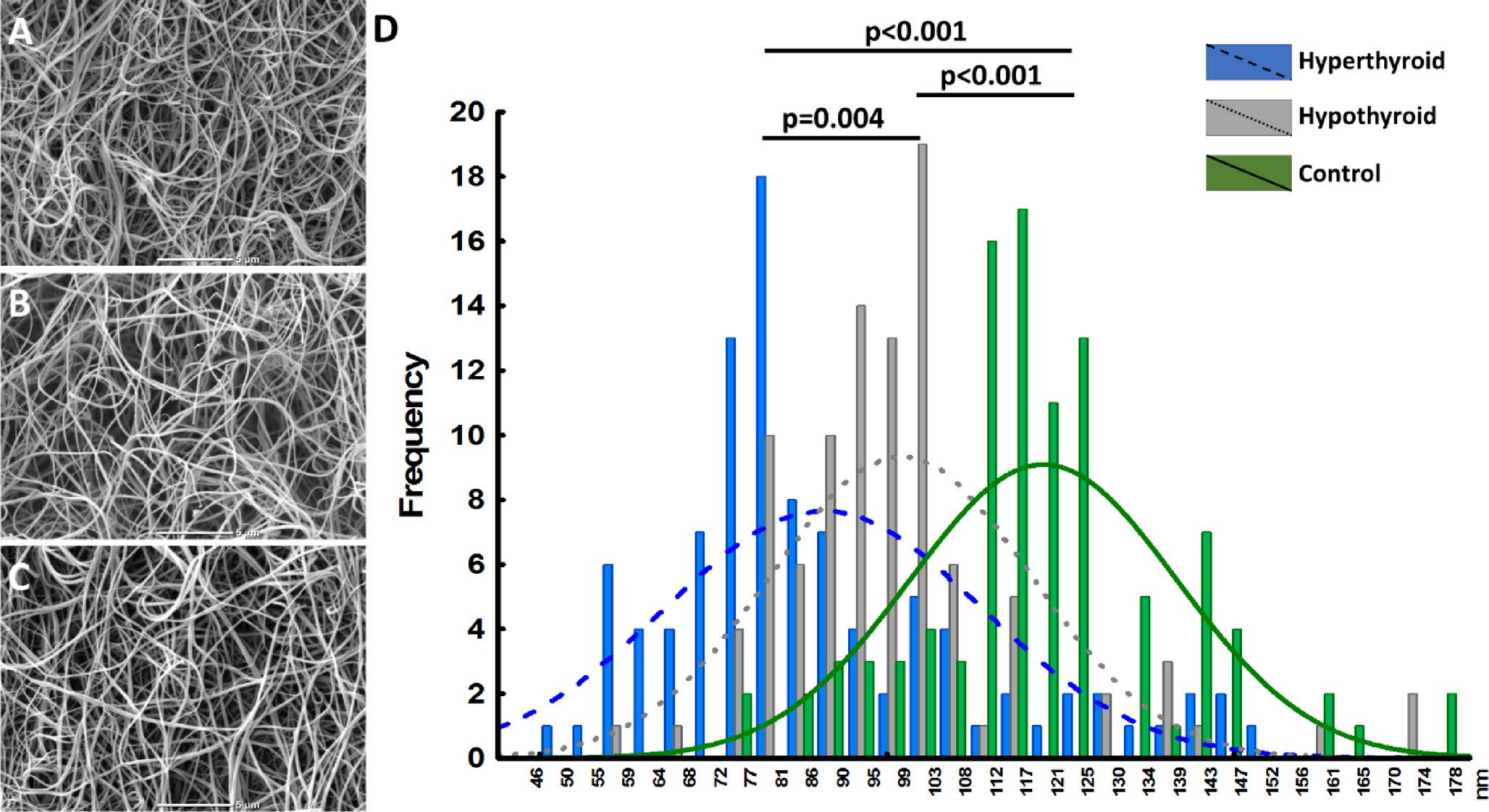
Representative scanning electron microscopy images of plasma fibrin clots of patients with hyperthyroidism (**A**), hypothyroidism (**B**) and controls (**C**) with similar plasma fibrinogen levels (3.5–3.6 g/L). Magnification, 5000x. Scale bar, 5 μm. Histogram of frequency of fibrin fiber thickness (**D**) in hyperthyroid, hypothyroid patients, and controls

Scanning electron microscopy images confirmed differences in fibrin clot structure between the groups (Fig. 3). The most prominent differences were between the hyperthyroid patients (Fig. 3A) and controls (Fig. 3C) with the fibrin clot structure characterized by thinner and more interconnected fibers within denser meshworks in the former group (Fig. 3D). Similar differences, though less pronounced, were noted between hypothyroid patients (Fig. 3B) and controls (Fig. 3C and D).

#### Fibrinolysis

**Fig. 4:**
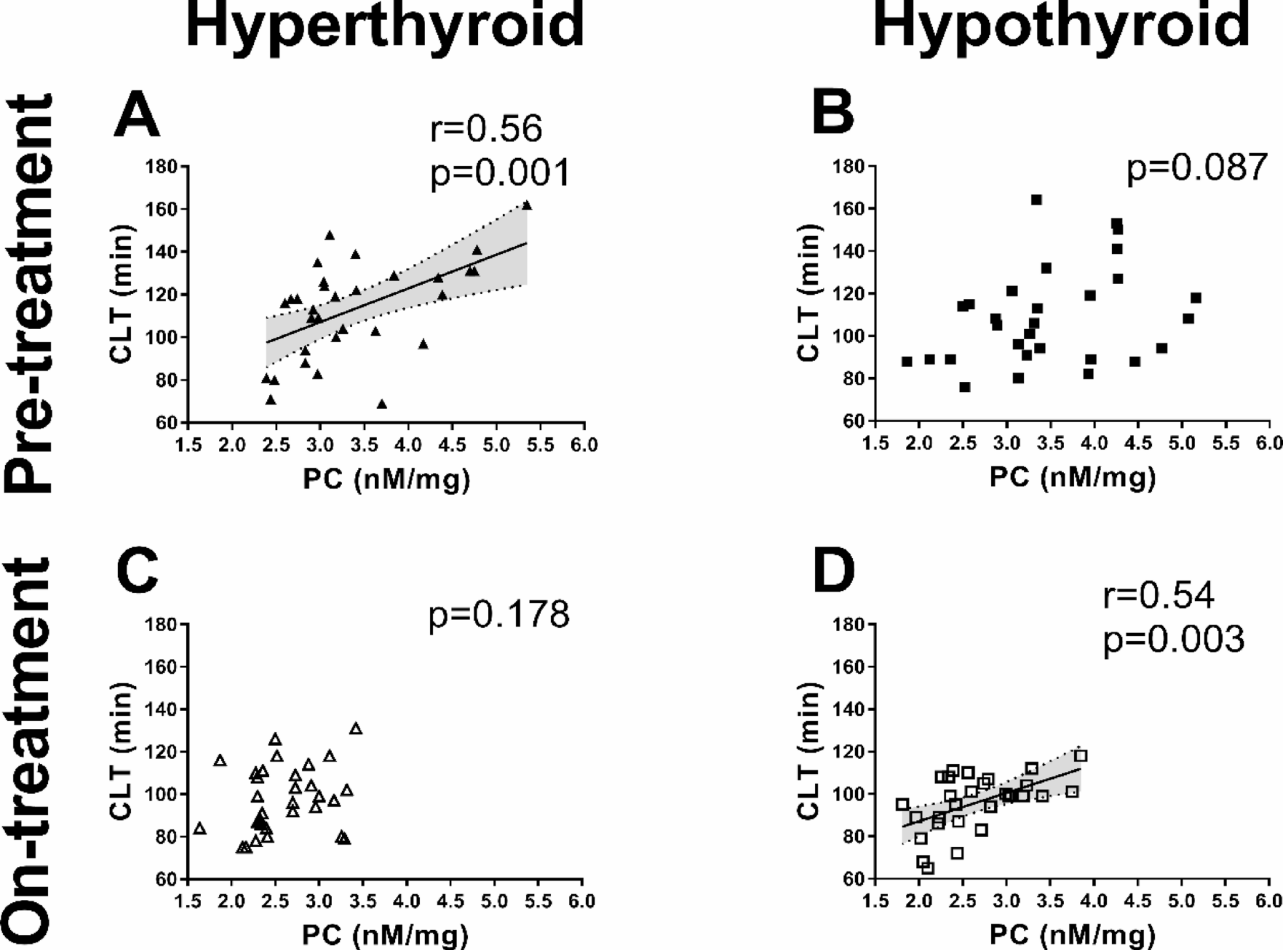
Correlations of protein carbonylation (PC) with clot lysis time (CLT) in hyperthyroid and hypothyroid patients before and on treatment

At baseline, patients with hyperthyroidism had 32.5% longer CLT (*p* < 0.001) compared with controls (Table 1). Among hypothyroid individuals CLT was prolonged by 26.8% (*p* < 0.001) in comparison to the control group (Table 1). In hyperthyroid patients PC correlated positively with CLT (Fig. 4A) and tended to be higher in hypothyroid subjects with prolonged CLT (*p* = 0.087, Fig. 4B).

Regarding fibrinolysis proteins, PAI-1 antigen was higher in hyper- and hypothyroid groups (by 151.1% and 100%, respectively, both *p* < 0.001) compared to controls (Table 1). Both TAFI antigen and activity were higher in hyperthyroid individuals by 22.2% (*p* < 0.001) and 45.3% (*p* < 0.001), respectively, and in hypothyroid patients by 17.8% (*p* < 0.001) and 34.9% (*p* < 0.001), respectively, compared to controls (Table 1). No associations of PC with PAI-1 and TAFI antigen/activity, or plasminogen were found in both patient groups (all *p* > 0.05).

**Table 1:** Patient and control characteristics at baseline and on treatment

Variable	Hyperthyroid patients (*n* = 31)			Hypothyroid patients (*n* = 29)			Controls (*n* = 29)
	Before treatment	On treatment	*p*-value	Before treatment	On treatment	*p*-value	
Age, years	45.00 (24.00–54.00)		-	50.00 (36.00–59.00)		-	47.00 (39.00–60.00)
Female, n (%)	27 (87.1)		-	23 (79.3)		-	24 (82.8)
BMI, kg/m^2^	24.41 (22.15–26.53)		-	26.14 (24.03–29.34)		-	24.60 (21.80–30.25)
Current smokers, n (%)	5 (16.1)		-	4 (13.8)		-	6 (20.7)
Hypertension, n (%)	5 (16.1)		-	7 (24.1)		-	5 (17.2)
CAD, n (%)	1 (3.2)		-	1 (3.4)		-	3 (10.3)
Diabetes mellitus, n (%)	5 (16.1)		-	0 (0)		-	2 (6.9)
**Laboratory variables**							
TSH, µIU/mL	0.005 (0.005–0.060)	2.64 (1.20–3.80)	< 0.001	9.26 (7.25–18.02)	2.60 (1.95-4.00)	< 0.001	2.05 (1.42–3.08)
FT4, pmol/L	29.12 (23.00-43.12)	14.01 (12.40-15.29)^a^	< 0.001	10.33 (9.18–12.21)^b^	14.80 (14.13–16.46)	< 0.001	-
Glucose, mmol/L	5.20 (4.80–5.84)	4.80 (4.29–5.52)	0.032	5.00 (4.77–5.65)	4.74 (4.25–5.81)	0.144	4.70 (4.45–4.85)
Creatinine, µmol/L	56 (46–63)	63 (54–70)	0.002	69 (57–75)	67 (65–76)	0.819	67 (60–78)
CRP, mg/L	1.14 (0.36–2.44)	1.40 (0.70–2.10)	0.829	0.88 (0.44–1.76)	1.01 (0.70–1.90)	0.265	1.40 (0.96–2.91)
LDL-C, mmol/L	2.08 (1.50–2.80)	2.82 (2.20–3.70)	< 0.001	3.10 (2.45–4.10)	3.10 (2.75–3.80)	0.151	2.45 (2.19–3.09)
Carbonyl Protein, nM/mg	3.11 (2.83–3.84)	2.50 (2.30–2.96)	< 0.001	3.34 (2.88–4.26)	2.56 (2.25–3.04)	< 0.001	2.85 (2.66–3.21)
**Hemostatic variables**							
Fibrinogen, g/L	3.77 ± 0.86	3.15 ± 0.69	< 0.001	3.56 ± 0.81	3.08 ± 0.73	0.003	3.36 ± 0.85
Peak thrombin, nM	274.29 ± 47.32	259.55 ± 45.83	0.007	273.59 ± 67.51	269.72 ± 46.89	0.602	253.00 ± 60.13
tPA antigen, ng/mL	7.18 (5.67–10.50)	6.13 (5.74–7.06)	0.126	8.10 (6.76–11.22)	6.33 (5.06–7.81)	0.001	10.70 (9.05–13.25)
PAI-1 antigen, ng/mL	33.90 (29.40–37.30)	28.10 (22.90–30.90)	< 0.001	27.00 (21.30–36.20)	26.30 (22.60-33.15)	0.092	13.50 (10.95–21.55)
PAI-1 activity, IU/mL	10.22 (8.41–12.26)	8.43 (6.81–9.93)	< 0.001	8.53 (6.40-10.59)	8.35 (6.23–10.69)	0.412	9.20 (7.50–10.00)
Plasminogen, %	98.06 ± 7.97	106.26 ± 12.02	0.004	103.97 ± 10.64	106.83 ± 13.33	0.408	104.07 ± 12.88
TAFI antigen, %	110 (100–119)	105 (87–116)	0.020	106 (101–121)	102 (86–111)	0.013	90 (84–100)
TAFI activity, µg/mL	33.13 (29.70-38.19)	30.95 (23.20-33.13)	0.039	30.75 (28.18–34.34)	26.40 (21.32–30.69)	0.004	22.80 (19.85–28.59)
**Fibrin clot variables**							
K_s_, 10^−9^ cm^2^	6.60 (6.10–7.20)	8.40 (7.20–9.20)	< 0.001	7.10 (6.20-8.00)	8.80 (7.25–9.45)	0.003	7.50 (7.00-7.95)
CLT, min	113.16 ± 22.77	97.87 ± 15.49	0.001	108.66 ± 22.91	95.93 ± 13.22	0.011	85.72 ± 15.92

^a^Available for *n* = 27

^b^Available for *n* = 28

Data are shown as median (interquartile range) or mean ± standard deviation. Abbreviations: BMI, body mass index; CAD, coronary artery disease; CLT, clot lysis time; CRP, C-reactive protein; FT4, thyroxine; K_s_, permeation coefficient; LDL-C, low-density lipoprotein cholesterol; PAI-1, plasminogen activator inhibitor-1; TAFI, thrombin-activatable fibrinolysis inhibitor; tPA, tissue-type plasminogen activator; TSH, thyroid-stimulating hormone

### On treatment

Among hyperthyroid individuals, 26 (83.9%) patients received thiamazole treatment and 6 (19.4%) RAI, while all cases of hypothyroidism were treated with LT4. Euthyroidism was achieved in all patients after 3 months. In the hyperthyroid group treatment led to 16.4% decrease in fibrinogen and 5.4% in peak thrombin, while hypothyroid group showed a 13.5% decrease in fibrinogen (Table 1).

As shown in Fig. 1, PC levels decreased by 19.6% in hyperthyroid and by 23.4% in hypothyroid patients (both *p* < 0.001). In both groups on-treatment PC was similar to values observed in controls (*p* = 0.089 and *p* = 0.205, respectively). On-treatment PC was associated with baseline PC in both hyperthyroid (*R* = 0.754; *p* < 0.001) and hypothyroid (*R* = 0.646; *p* < 0.001) individuals. Changes in fibrinogen observed at 3 months were not associated with on-treatment PC or reduction in this marker in treated hyper- or hypothyroid patients.

#### Fibrin clot permeability

In the hyperthyroid group, we observed 27.3% improved K_s_ as a result of restoring euthyroidism, while in the hypothyroid group, K_s_ increased by 23.9% compared to baseline (Table 1). No association of on-treatment PC with K_s_ was found in the hyperthyroid group after 3 months of therapy (Fig. 2C). However, on-treatment PC in hypothyroid subjects who restored euthyroidism correlated negatively with K_s_ (Fig. 2D).

#### Fibrinolysis

In the hyperthyroid group, treatment led to CLT shortened by 13.5% as a result of restoring euthyroidism and in the hypothyroid group, CLT shortened by 11.7% compared to baseline (Table 1). No association of on-treatment PC with CLT was found in the hyperthyroid group after 3 months of therapy (Fig. 4C), while in hypothyroid subjects who restored euthyroidism PC correlated positively with CLT (Fig. 4D).

In the hyperthyroid group, we noted approximately 17% reduced in PAI-1 antigen and activity as well as 8.4% higher plasminogen (Table 1). A slight decrease in TAFI antigen and activity was observed in this group (Table 1). Meanwhile, among hypothyroid patients a slight decrease in TAFI activity and antigen were found on therapy compared to pretreatment results (Table 1).

No associations of fibrinolysis inhibitors changes with PC on treatment were found in either patient group.

## Discussion

To our knowledge, the current study is the first to show that in hyperthyroidism and hypothyroidism elevated PC levels in circulating blood are associated with formation of more compact fibrin fiber networks, a risk factor for thromboembolism. Unexpectedly, impaired fibrin lysability has been associated with PC only in hyperthyroidism. With on-treatment thyroid hormone normalization and significant decreases in PC at 3 months, fibrin clot characteristics improved, but on-treatment PC levels correlated with clot permeability and lysability only in hypothyroid patients. The current study provides new insights into the intricate role of enhanced oxidative stress in the modulation of prothrombotic alterations to fibrin structure and function in thyroid disorders, indicating that the extent of carbonylation within diverse proteins modulates the final stage of blood coagulation in vivo also in hyperthyroidism and hypothyroidism.

Our study showed that individuals with hyperthyroidism and hypothyroidism exhibited higher PC concentrations compared to healthy controls, though in the former group the difference was a non-significant trend. Elevated PC in hyperthyroidism was demonstrated previously, among others, by Mseddi et al. [34], who reported more than 2-fold higher PC levels in 34 patients with Graves’ disease as compared to 65 healthy controls. In our study the difference was much smaller, which could possibly be attributed to distinct patient characteristics and differences in the assays used. Participants in the study by Mseddi et al. [34] were younger and PC levels were measured using a different protocol [35]. Elevated PC in hypothyroidism has also been demonstrated previously [20, 22, 36]. Since in the study by Chandra et al. [20] subjects with comorbidities, smoking habit, or taking any medications were not eligible, it is likely that those factors contributed to the magnitude of differences in PC levels between hypothyroid and normal subjects observed in available studies. Moreover, the different assays were used to measure PC in that study [20, 37] and ours. Taken together, the present study confirmed that PC content in circulating plasma was increased in thyroid dysfunction. Importantly, we demonstrated that on-treatment PC levels when thyroid function is restored become comparable to those in well-matched controls, suggesting that effective 3-month therapy may normalize the redox status.

The key novelty of our study is that higher PC levels are associated with reduced fibrin clot permeability in both hyper- and hypothyroid group, while plasma clot lysability, unrelated to PAI-1 or TAFI concentrations or activity, showed association with PC solely in the former group, suggesting the structural alterations to fibrin provoked by hyperthyroidism affect susceptibility to lytic degradation to a larger extent. Surprisingly, in patients with thyroid disorders we observed increased PAI-1 levels, without any changes in its activity. However, PAI-1 carbonylation has been demonstrated to influence its functional activity and structural stability [38], therefore carbonylated PAI-1 may exhibit reduced activity, and we hypothesize that it could trigger a compensatory mechanism leading to an increase in its overall concentration observed in our cohort.

Fibrinogen is the key determinant of fibrin clot structure and density of networks [39]. At the molecular level, PC introduces carbonyl groups into the fibrinogen molecule as a result of oxidative modifications, particularly affecting amino acid residues such as methionine and tyrosine [40]. These alterations compromise the structural integrity of the α domain, essential for lateral fibrin aggregation [41]. In our study, PC did not correlate with fibrinogen concentrations though fibrinogen is known to be 20-fold more sensitive to oxidation than albumin [42]. This supports the view that carbonylation may affect specific functions of various proteins forming clots (almost 500 in proteomic analysis) [43], but the alterations do not alter the results of the Clauss assay in thyroid dysfunction. In vitro studies have confirmed that at the exposure to low ROS concentrations encountered in vivo (equivalent to < 0.2 mM Fe^2+^/H_2_O_2_), fibrinogen clotting is unaffected [44] and consequently there is no association of PC (determined in our assay) with fibrinogen measured using routine clotting assays in most disease states [9, 13, 14, 45]. Furthermore, carbonylation of proteins involved in fibrinolysis, for instance plasminogen, cannot be excluded as a contributor to hypofibrinolysis as shown in patients with pulmonary embolism [11].

Surprisingly, decreased PC levels observed following normal thyroid function restoration were associated with improved clot permeability and lysability solely in treated hypothyroid patients. A few potential hypotheses could be put forward. Since ROS are a physiological element of human biochemistry, only after exceeding certain levels of ROS generation they could have a higher chance of causing unfavorable changes in fibrin properties, proportional to PC levels. Additionally, hyperthyroidism was shown to increase protein catabolism leading to faster elimination of modified proteins, including carbonylated ones [46]. It might be also speculated that hypothyroid patients with more comorbidities in particular cardiovascular risk factors and disease, known to unfavorably affect clot properties, for instance age, obesity, hypercholesterolemia [6, 32] exhibit the significant associations, which are abolished in relatively “healthier” hyperthyroid individuals. Hypothyroidism remains often undiagnosed for a longer period [47] leading to disturbed oxidative balance of prolonged duration, which may cause more lasting changes in fibrinogen function that could be observed after 3 months. It might be hypothesized that with time and further restoration of redox balance, reduced PC will lose association with fibrin clot properties, however this concept requires further research to assess the associations during longer follow-up time.

Several study limitations should be acknowledged. A key limitation is a limited sample size. Therefore, our report should be considered as preliminary, hypothesis-generating research and the data interpretation should be approached cautiously. In particular, correlations in the patient groups should be treated with extreme caution due to limited statistical power. Associations do not necessarily mean the cause-effect relationship. Exact mechanisms behind PC-related changes in fibrin clot structure and function, particularly assessment of purified fibrinogen carbonylation and oxidant/antioxidant balance were beyond the scope of the present study.

In conclusion, the current study demonstrates that posttranslational oxidative protein modification, carbonylation, affects fibrin clot structure and lysability in hyperthyroidism and hypothyroidism in a differential manner. In both thyroid disorders elevated PC is associated with formation of more compact fibrin clots, typical of increased thrombotic risk. The impact of PC on fibrinolysis shows a different regulation in hyperthyroidism and hypothyroidism both before and after restoring euthyroidism. Our findings, if validated in future studies with larger sample sizes, might suggest the potential for oxidative stress-targeted interventions to reduce thrombotic risk in thyroid dysfunction, as well as potential benefits from anti-thrombotic agents in severe thyroid disorders.

## Data Availability

No datasets were generated or analysed during the current study.
